# Draft genome sequence of *Pseudomonas aeruginosa* Pa047c, a pan-resistant strain isolated from an ICU in Manaus, Amazonas

**DOI:** 10.1128/mra.00571-24

**Published:** 2025-12-30

**Authors:** Daniel Jesus de Figueiredo, Eraldo Ferreira Lopes, Vanda Santana Queiroz Dini, Fernanda de Almeida Batalha, João Victor De Melo Verçosa, Alessandra Pontes Cavalcanti, Ivanildes dos Santos Bastos, Paulo Afonso Nogueira, Ruth Moura de Souza, Natália Lopes, Paula Taquita Serra, Adolfo José da Mota, Patrícia Puccinelli Orlandi

**Affiliations:** 1 Laboratório de Diagnóstico e controle de doenças infecciosas na amazônia (DCDIA), Instituto Leônidas & Maria Deane, Fiocruz Amazônia 560875 , Manaus, Amazonas, Brazil; 2 Programa De Pós Graduacão em Biologia da Interação Patógeno Hospedeiro, Instituto Leônidas & Maria Deane, Fiocruz Amazônia 560875 , Manaus, Amazonas, Brazil; 3 Laboratório de Microbiolgia, Instituto de Saúde e Biotecnologia de Coari (ISB-Coari), Universidade Federal do Amazonas (UFAM) 67892, Coari, Amazonas, Brazil; 4 Aluna de doutorado do Programa de Pós Graduação em Imunologia Básica e Aplicada, Universidade Federal do Amazonas (UFAM) 67892, Manaus, Amazonas, Brazil; 5 Laboratório de Biodegradação, Faculdade de Ciências Agrárias, Universidade Federal do Amazonas (UFAM) 67892, Manaus, Amazonas, Brazil; 6 Instituto Aggeu Magalhães (IAM), Fiocruz 92923 , Recife, Brazil; University of Maryland School of Medicine, Baltimore, Maryland, USA

**Keywords:** *Pseudomonas aeruginosa*, antibiotic resistance, efflux system, Amazonia

## Abstract

*Pseudomonas aeruginosa* is currently considered a high-risk bacterium by the World Health Organization. Here, we present the draft genome of the pan-resistant *P. aeruginosa* Pa047c strain isolated from an intensive care unit from a hospital in the city of Manaus, Amazonas, Brazil.

## ANNOUNCEMENT

Bacterial antimicrobial resistance (AMR) is one of the greatest problems in medicine, negatively impacting the treatment time, prognosis, increasing hospital costs, and morbimortality (1). Several threatening microorganisms to humans are monitored by the World Health Organization; among these, *Pseudomonas aeruginosa* is classified as high priority due to its resistance capacity to a wide variety of antibiotics, from various classes, such as β-lactams, aminoglycosides, fluoroquinolones, tetracyclines, and chloramphenicol (2–4).

Here are reported the genomic findings of *P. aeruginosa* Pa047c, a pan-resistant strain isolated from the oral cannula of a patient admitted to a public hospital in Manaus-AM (the study was approved by the appropriate ethics committee). Samples were routinely collected from patients and hospital environments in a surveillance project that assessed the occurrence of resistant *Pseudomonas* in hospitals. Those samples were cultured on Pseudomonas isolation agar at 37°C for 24 hours. Biochemical characteristics such as positive cytochrome oxidase, motility, and growth at 42°C were used as criteria for further analyses, such as identification by 16S rRNA sequencing and AMR, following the Clinical and Laboratory Standards Institute standards (5, 6). 

Pa047c strain was selected for genomic analysis because it showed resistance to all tested antibiotics, as well as associations: Aminoglycosides (Amikacin, Gentamicin, and Tobramycin); Antipseudomonal penicillins (Piperacillin associated with Tazobactam); Third-generation cephalosporins (Ceftazidime); Fourth-generation cephalosporins (Cefepime); Carbapenems (Imipenem and Meropenem); Monobactams (Aztreonam); Second-generation fluoroquinolones (Norfloxacin and Ciprofloxacin); Third-generation fluoroquinolones (Levofloxacin); and Polymyxins (Polymyxin B).

The total DNA for genomic sequencing was prepared from 1 mL of fresh Pa047c inoculum, grown on an Luria Bertani (LB) broth overnight at 150 rpm at 37°C (7). The DNA was lyophilized and sent to GenOne: Soluções em Biotecnologia (Rio de Janeiro, RJ, Brazil) for sequencing of 3 Gb PE150 (Q30 >85%) in an Illumina Hiseq4000, using “NEBNext Ultra DNA Library Prep Kit,” according to the manufacturer’s recommendations, which yielded 23,743,518 raw reads.

The quality assessment was obtained using FastQC v.0.11.8 (8); adapter removal and quality filtering were accomplished using Trimmomatic v.0.39 (9), and the assembly was carried out through Unicycler v.0.4.8.0 (10, 11), using selecting bridging mode as normal, with 10 k-mers steps during assembly, 0.2 as lowest k-mer size as a fraction of read length, and excluding contigs lower than 100 bp. Default parameters were used for all software unless otherwise specified.

The assembly generated 115 contigs, with a coverage of 96.18% in relation to the reference genome GCF_002997005.1 and an N50 of 269,094 bp. The draft length was 6.9 Mb with a GC content of 65.9%. The annotation, performed by NCBI Prokaryotic Genome Annotation Pipeline (12), mapped 6,902 coding sequences and 6,686 genes, 3 genes for rRNA, 61 genes for tRNA, and 1 gene for tmRNA.

This work reports the Pa047c draft genome, whose annotation revealed β-lactamase resistance genes, including a few for broad-spectrum antibiotics (ESBL), such as OXA-50, Int1, and VIM. This strain also has nine efflux systems, including MexAB-OprM (13, 14). A missense mutation (cV126E) was also noted in the mexR gene, a negative regulator of the MexAB-OprM operon (15).

## Data Availability

The genome draft is available under GenBank accession number JACTAF010000000 and SRX9049814. The raw data were deposited in the SRA with the accession number PRJNA660159.
